# Sustainability and environmental stewardship are ethical responsibilities for nurses: a commentary

**DOI:** 10.1177/17449871251361447

**Published:** 2025-10-29

**Authors:** Eva-Lena Einberg

**Affiliations:** Senior Lecturer, Kristianstad University, Kristianstad, Sweden

**Commentary to:** Sustainability and nursing: An integrative literature review (de Diego Codero et al., 2025).

Climate change represents the greatest threat to human health (WHO, 2025). The health sector must therefore intensify its efforts both to adapt to climate change and to reduce its greenhouse gas emissions. All health professionals have a role to play in addressing these challenges. Nurses, as the largest group of health professionals, possess the potential to drive meaningful change and are often willing to contribute – provided they are empowered to do so. According to the International Council of Nurses (ICN, 2021), sustainability and environmental stewardship are ethical responsibilities for nurses.

This study (de-Diego-Cordero et al., 2025) presents an integrative review with the aim of understanding the role of nursing and its importance within the health field for sustainability in global healthcare. Inadequate adaptation to climate change increases the vulnerability of health systems. The health sector bears a particular responsibility, as it is tasked with promoting health and preventing illness, while simultaneously contributing significantly to greenhouse gas emissions and managing the health impacts of climate change. Climate-related challenges that will strain healthcare services include heatwaves, flooding, limited access to clean water, and the spread of infectious diseases. These developments will, in turn, increase the demand for nursing, making nurses’ competencies increasingly relevant.

de-Diego-Cordero et al. (2025) emphasise the unique role of nurses as key actors in achieving the 2030 Agenda for Sustainable Development. Nurses are central to sustainability efforts due to their core responsibilities in caring for individuals, promoting health and preventing disease. The Sustainable Development Goals (SDGs) offer a framework through which nurses can apply their knowledge to enhance health and well-being. Moreover, nurses are trained to work holistically and often serve as a bridge between communities and healthcare systems, giving them valuable insight into how policy decisions affect individuals. Despite this expertise, nurses’ perspectives are often overlooked by policymakers.

de-Diego-Cordero et al. (2025) also explore the concept of the circular economy as a potential pathway towards sustainable healthcare, though it acknowledges the resistance such models face within the current economic paradigm. Despite challenges such as workforce shortages and limited opportunities for nurses to influence policy, nurses possess the knowledge and skills necessary to lead the transition toward more sustainable and resource-efficient care. Their contributions span both clinical practice and leadership roles. The authors propose strategies to overcome barriers to integrating sustainability into nursing practice.

Education plays a critical role in empowering nurses to engage in sustainability efforts. To recognise their value in this domain, sustainability must be emphasised throughout nursing education. Curricula should include content on systems thinking, the interconnection between local and global health and the development of critical thinking skills, information literacy and participatory decision-making. Concepts such as social justice and global citizenship should also be incorporated.

In addition to identifying challenges and proposing solutions, the study by de-Diego-Cordero et al. (2025) highlights ongoing efforts to elevate the visibility and relevance of nursing knowledge in sustainability work. Nurses can engage with sustainability issues at local, regional and global levels through professional organisations and associations. One example is the Swedish Society of Nursing’s working group on health and climate, which has identified key areas where nurses’ expertise and commitment are particularly impactful. These areas, along with practical examples, are outlined in a publication available to nurses (Einberg, et al., 2025; The Swedish Society of Nursing, 2024) to support their sustainability initiatives.

In conclusion, de-Diego-Cordero et al. (2025) demonstrates that nurses’ knowledge is vital to advancing sustainability in healthcare. However, further efforts are needed to enable nurses to fully contribute. Nurses should be more actively involved in decision-making processes within the health sector and society at large, to help mitigate healthcare’s impact on the climate and to support the development of climate-resilient health systems. Nursing education must evolve to equip future nurses with the competencies required to meet these challenges and to lead sustainability efforts effectively.
